# Carboxymethyl-Cellulose-Containing Ag Nanoparticles as an Electrochemical Working Electrode for Fast Hydroxymethyl-Furfural Sensing in Date Molasses

**DOI:** 10.3390/polym15010079

**Published:** 2022-12-25

**Authors:** Nashi K. Alqahtani, Tareq M. Alnemr, Faisal A. Shulaybi, Hisham Abdelmonem Mohamed, Mohamed Gouda

**Affiliations:** 1 Date Palm Research Center of Excellence, King Faisal University, P.O. Box 400, Al-Ahsa 31982, Saudi Arabia; 2 Department of Food and Nutrition Sciences, College of Agricultural and Food Sciences, King Faisal University, Al-Ahsa 31982, Saudi Arabia; 3 Date Palm Research Center Al-Ahsa, Ministry of Environment, Water and Agriculture, Al Mubarraz 36321, Saudi Arabia; 4 Department of Chemistry, College of Science, King Faisal University, Al-Ahsa 31982, Saudi Arabia

**Keywords:** nanocomposite, AgNPs, HMF sensor, electrochemical sensor, voltammetric analysis

## Abstract

Novel biosensors based on carboxymethyl cellulose extract from date palm fronds containing Ag nanoparticles as an electrochemical working electrode for fast hydroxymethylfurfural (HMF) sensing in date molasses were prepared. The morphological, structural, and crystallinity characteristics of the prepared Ag@CMC were described via SEM, DLS, TEM, and XRD. In addition, Raman spectroscopy and UV–VIS spectroscopy were performed, and thermal stability was studied. The investigated techniques indicated the successful incorporation of AgNPs into the CMC polymer. The sensing behavior of the prepared AgNPs@CMC electrode was studied in terms of cyclic voltammetry and linear scan voltammetry at different HMF concentrations. The results indicated high performance of the designed AgNPs@CMC, which was confirmed by the linear behavior of the relationship between the cathodic current and HMF content. Besides, real commercial samples were investigated using the novel AgNPs@CMC electrode.

## 1. Introduction

Furanic compounds can be naturally produced by the dehydration of natural sugars, especially at low pH, or by heating of carbohydrate foods [1,2]. 5-Hydroxymethylfurfural (HMF) is one of the most impacted by-products of non-enzymatic reactions and can be produced from sucrose or fructose [3,4] or glucose derivatives [5,6] or cellulose [7]. The chemical existence of HMF has been recorded in several natural or industrial foods, such as honey, biscuits, and jams, in addition to some pharmaceutical syrups [8]. Therefore, the HMF content in natural or commercial food should be analyzed for quality control measurements [9]. More content of HMF in honey indicates overheating and freshness loss [10]. In short, the fast measurement of the HMF content is one of the important indicators of quality deterioration in commercial or naturally modified food.

Variable traditional techniques have been developed, such as capillary electrophoresis with diode array detection [11], HPLC with UV detection [12], ion-exclusion chromatography with UV detection [13], capillary zone electrophoresis–tandem ion trap mass spectrometry [14], and different types of HPLC [15], to investigate the HMF concentration in different commercial foods. The demerits of the traditional methods are using harmful chemicals as solvents (e.g., methanol in the case of HPLC) or reactants, which has a negative impact on the environment or human health.

Electrochemical methodology has high potential because of several merits, including a quick and easy response, in addition to high sensitivity and environmental safety [16,17]. Previously, the use of these techniques was reported in order to analyze dopamine [18], nitrite [19], and Hg [20] and recently reported in order to analyze heavy metals [21], zearalenone residues [22], paracetamol [23], sulfonamides [24], chloramphenicol [25], paraquat in food samples [26], vitamin C [27], and bisphenol A in plastic products [28]. Therefore, in this work, the electrochemical technique was used to analyze the HMF concentration by using novel electrodes based on nanoparticles and polymer content.

Recently, electrochemical approaches to hydroxymethylfurfural sensing were studied. Co-electrodeposited Cu-Ni microparticles were presented for electrocatalytic oxidation of hydroxymethylfurfural [29]. The obtained data based on Cu-Ni microparticles showed a linear dependency within the range of 0.4–10 × 10^−3^ M, with a detection limit of 3.51 × 10^−6^ M. Additionally, 3D graphene-like surface (3DGrls) was introduced for the electrochemical sensing of hydroxymethylfurfural and showed a good linear response in a concentration range of 0.35~116 × 10^−6^ mol/L, with a low detection limit of around 0.099 × 10^−6^ mol/L [30]. In addition, the deposition of Co/Al or Fe-layered double hydroxides (LDHs) was introduced to electro-oxidize hydroxymethylfurfural [31]. The LDH materials have an interesting structure that has been reported for many chemical and electrochemical applications [32,33]. The reported approach based on LDHs focuses on potentiodynamic cathodic reduction. Co/Al-LDH has better performance as an electrochemical sensor compared with Co/Fe-LDH [31]. Silver microdendrites were recently introduced to improve the electro-analytical efficiency of screen-printed electrodes [34]. The cathodic peak of hydroxymethylfurfural was found at −1.48 V, less than that observed from the bare screen-printed electrode. Furthermore, the silver electrode had a better current response than the pristine electrode, affirming that Ag not only has enhanced electrocatalytic characteristics but also increases electrical conductivity and has a better electro-active surface area. The current response linearly went up with more hydroxymethylfurfural in the range of 3–100 ppm and a detection limit of around 1.0 ppm. Previously, Ag nanoparticles have been applied in different chemical applications, including photocatalysis [35], degradation of sulfamethoxazole [36], electrochemical CO_2_ to CO conversion [37], alkaline direct ethanol fuel cells [38], flexible organic electrochromic devices [39], and biochemical applications [40]. Therefore, in this work, Ag was introduced to be incorporated into the polymer content to improve the electroanalytic response of the polymer used.

Polymers were incorporated with several nanoparticles to improve their performance [41]. Polymers coming from natural sources have environmental and renewable advantages. Examples of these polymers include chitosan, starch, and gum, in addition to carboxymethyl cellulose (CMC), which was used in this study. CMC was introduced as a substrate with different composites for electrochemical detection of 1,5-anhydroglucitol [42] and selective detection of hydrogen peroxide [43]. In this work, AgNPs were incorporated into the CMC matrix and then applied as an electrochemical electrode for HMF sensing. The prepared Ag@CMC was studied physiochemically to confirm its morphology, crystallinity, and thermal analysis. After that, cyclic voltammetry and linear scan voltammetry were performed at different known HMF contents, followed by an analysis of real samples containing HMF.

## 2. Materials and Methods

### 2.1. Materials

Date palm fronds were primarily dried in daylight and then pulverized in a mixer to obtain a particle size of 500 μm. The dried powders were then soaked in isopropanol in a Soxhlet apparatus for 18 h at 70 °C. The powders were removed from the Soxhlet apparatus and then dried in an oven at 70 °C for 24 h. The dried-oil-removed date palm frond powders (25 g) were treated with 758 mL of NaOH (7.5%) under stirring for 1 h at room temperature (25 °C). The treated powder samples were filtered and washed with an ethanol/water mixture (90:10) several times to remove sodium hydroxide. The hemicellulose-free date palm frond powders were dried in an oven at 70 °C for 24 h. To remove lignin, 3 g of hemicellulose-free date palm frond powders were added separately to a flask containing 100 mL of distilled water, 20 mL of CH_3_COOH acid, and 3 g of NaClO_2_. The mixture was then stirred at 85 °C for 2 h. The lignin-free date palm frond powders were filtered and washed with an ethanol/water mixture (90:10). Finally, the washed lignin-free date palm fronds were dried in an oven at 70 °C for 24 h.

### 2.2. Preparation of Carboxymethyl Cellulose Extract

The following procedure was used to prepare carboxymethyl cellulose (CMC): Cellulose (1 mole) was treated in a glass container with 2 M sodium hydroxide at 80 °C for 10 min, after which 1 M monochloroacetic acid was added. The mixture was mechanically agitated for 10 min before being held in an 80 °C water bath for 3 h. After the reaction period, the reaction product was cleaned using a Soxhlet apparatus for 24 h with a 70:30 isopropanol/water solution. The sample after washing was dried in a 70 °C oven. CMC was produced with a degree of substitution (D.S) of 2.7.

### 2.3. Synthesis of AgNPs@CMC Nanocomposites

Nanocomposites made of AgNPs and CMC were prepared as follows: Briefly, 5 mL of 0.1 M silver nitrate solution and 50 mL of acetate buffer solution (pH 6–6.5) were put to a glass-stoppered vial containing 0.1 g of CMC. For 24 h, the mixture was then shaken irregularly at room temperature. Silver ions that had been chelated with CMC were added to a reducing solution comprising 10 mL of H_2_O_2_ (35%) and 4 g/L of NaOH at a pH of 9.4. An ultrasonic stirrer agitated the mixtures for 2 hours. The finished AgNPs@CMC nanocomposites were filtered out, carefully washed with water, and then dried for 20 min at 60 °C.

### 2.4. Characterization

The extracted cellulose was characterized using a thermogravimetric analyzer (TGA; TA instrument model Q 500-USA) to investigate its thermal stability. The surface morphology of the extracted cellulose was characterized using a JOEL JXA-840 scanning-electron-microscope-type electron probe microanalyzer. The crystal structure of cellulose extracts and silver oxide/carboxymethyl cellulose nanocomposites was analyzed using XRD with a D/max-IV diffractometer with Cu K*α* radiation and a scanning electron microscope, and the prepared nanoparticle solution was characterized using UV–VIS spectroscopy.

### 2.5. Electrochemical Determination of HMF

The Autolab PGSTAT302N potentiostat/galvanostat was used to conduct electrochemical measurements. Briefly, 5 mg of the prepared nanocomposites (AgNPs@CMC) was spread in 80 μL of Nafion solution (5%), 200 μL of ethanol, and 800 μL of distilled water to obtain electrodes. In a three-electrode batch cell, HMF electrochemical conversion was assessed using linear voltammetry (LV) tests carried out at 20 mV/s in phosphate buffer solution (pH 9). pH is an important factor in electroanalytical techniques, which should be at the optimum condition. In acid solutions, acid-catalyzed hydrolysis converts HMF into gamma-hydroxyvaleric acid and gamma-valerolactone. Additionally, a strong alkaline medium can affect the chemistry of HMF by basic hydrolysis. Therefore, the pH was adjusted to 9. The working electrode was a AgNPs@CMC/glassy carbon electrode (GCE), the reference electrode was a saturated calomel electrode (SCE), and the counter-electrode was a platinum wire. All electrochemical tests were carried out in a typical three-electrode cell configuration. Prior to conducting the voltammetry studies for the sensing of HMF, the solution was purified for 10 min with high-purity nitrogen gas, and the gas was kept flowing above the solution to preserve the nonoxygen conditions. The IM6E impedance measuring device provided EIS (Zahner-Elektrik, Thüringer, Kronach – Gundelsdorf, Germany). An open-circuit potential with an amplitude of 50 mV and a frequency range of 0.01 Hz to 100 kHz was used to sustain the 5 mM [Fe(CN)_6_]^3−/4−^ solution containing 0.1 M KCl.

### 2.6. LV Determination of Real Samples

Three samples of date molasses were bought from a nearby grocery. Next, 0.50 g of the molasses was sonicated for 30 min after being mixed with 12 mL of water. The dispersion was stirred for 20 min, and then, 5 mL of new 0.15 M oxalic acid was added. The dispersion was then heated in a water bath (100 °C) for 25 min. After that, the cooling solution underwent pretreatment using conventional procedures [44,45]. A portion of the pretreatment sample was introduced to an electrochemical cell for LV measurement, and PBS (pH 9.18) was then added to make a final 10 mL solution. The content of HMF was also determined using the standard curve.

## 3. Results and Discussion

### 3.1. Material Characterization

#### 3.1.1. XRD Analysis

**Figure 1:**
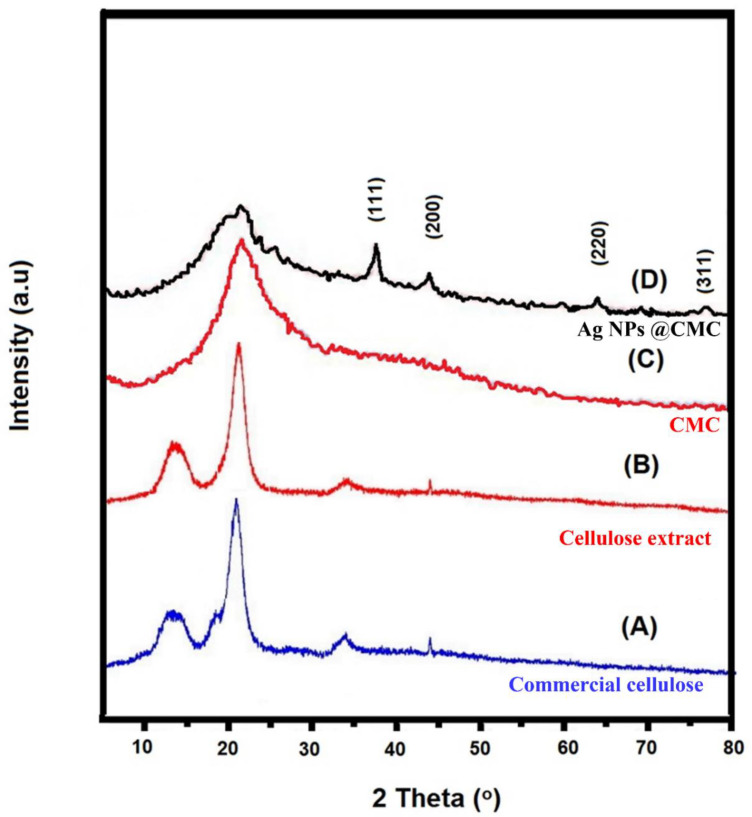
XRD of (**A**) commercial cellulose, (**B**) cellulose extract, (**C**) CMC extract, and (**D**) AgNPs@CMC.

XRD analysis was used to investigate the crystallinity of the prepared Ag/polymer composite [46]. XRD investigation for commercial cellulose, cellulose extract, CMC extract, and AgNPs@CMC was carried out, as displayed in Figure 1, at 2θ = 5° to 80°. The extracted cellulose had the same XRD peak positions as the commercial cellulose, which confirms the successful extraction of cellulose. Both commercial and extracted cellulose had amorphous characters, which are indicated by the broad peaks at 2θ around 13.08° and 21.34°. After the formation of CMC, the XRD peaks changed to be more amorphous. After that, the XRD 2θ profile had a broad peak of amorphous CMC beside the characteristic peaks of Ag nanoparticles (AgNPs) and was in accordance with the reported XRD card (JCPDS 004-0783) [47,48]. The 2θ positions were seen at 38.9°, 44.7°, 64.9°, and 78.1°, which could be due to the planes of (111), (200), (220), and (311), respectively. These XRD data confirm the successful design of cubic AgNPs (fm3m-225) in the CMC structure containing an amorphous part from CMC and a crystalline part from the AgNPs’ side.

#### 3.1.2. Surface Morphology

**Figure 2:**
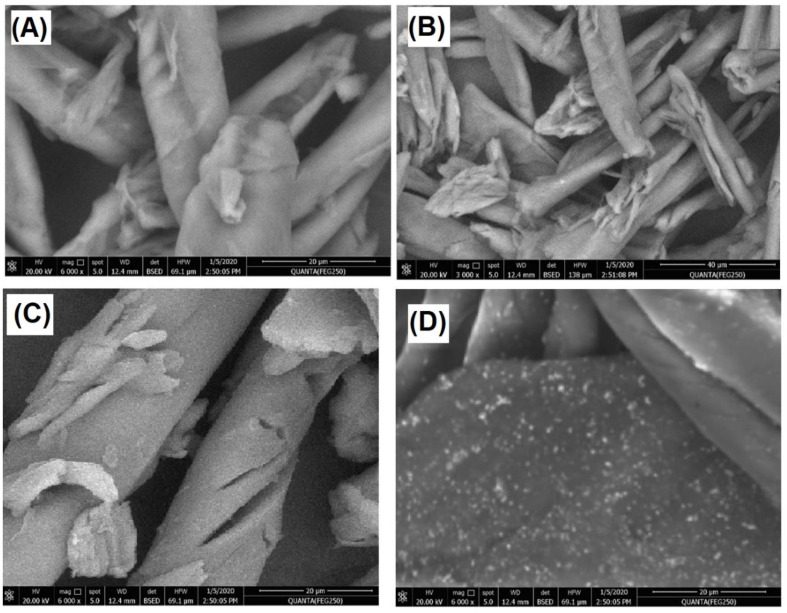
SEM images of (**A**) commercial cellulose, (**B**) cellulose extract, (**C**) CMC extract, and (**D**) AgNPs@CMC.

The morphology of commercial cellulose, cellulose extract, CMC extract, and AgNPs@CMC was inspected, as described in Figure 2A–D, respectively. As seen from the SEM images, a fibrous morphology could be observed for commercial cellulose, cellulose extract, and CMC extract, which could be expected from previous studies [49]. However, the SEM image of the modified AgNPs@CMC had a composite morphology between nanoparticles and a fibrous morphology. Therefore, the AgNP material was confirmed to have a nanoscale size, as shown in Figure 2D. Finally, XRD combined with SEM confirmed the incorporation of crystalline AgNPs into the CMC structure.

#### 3.1.3. TEM and DLS Analysis

**Figure 3:**
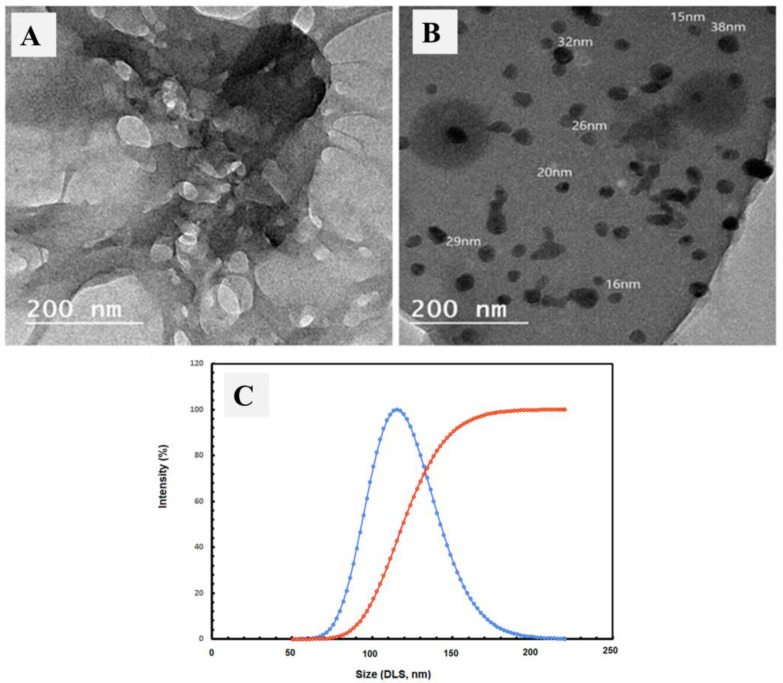
TEM images of (**A**) carboxymethyl cellulose extract, and (**B**) AgNPs@CMC extracts and DLS analysis of AgNPS@CMC extract (**C**).

The TEM images were inspected for the carboxymethyl cellulose extract and AgNPs@CMC extract, as shown in Figure 3A and 3B, respectively. The TEM image of carboxymethyl cellulose extract showed no clear nanoparticles, which could be due to the fibrous morphology, as shown in the SEM investigation. In contrast, Figure 3B shows obvious nanoscale-size particles, which could be attributed to Ag particles, besides the morphology of the CMC matrix. Additionally, dynamic light scattering (DLS) analysis of the AgNPs@CMC extract was performed to confirm the particle size distribution. The obtained size of the prepared particles was bigger than what was previously observed through SEM or TEM, which has a scientific reason (DLS investigation measures the hydrodynamic diameter). The maximum particle size was measured at 109 nm for AgNPs@CMC, which indicates the nanoscale size of the Ag particles formed at the CMC matrix. In short, the DLS investigation confirmed the AgNPs’ loading to form AgNPs@CMC material.

#### 3.1.4. Raman Spectroscopy

**Figure 4:**
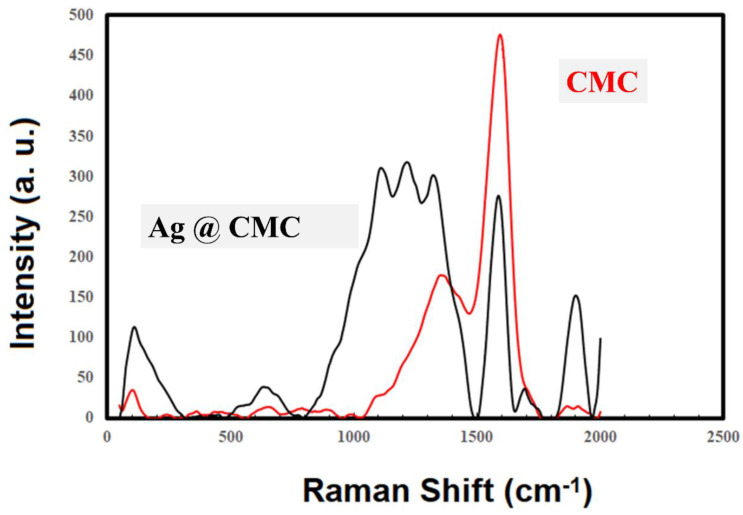
Raman spectroscopy of CMC and AgNPs@CMC.

Raman spectroscopy was performed for the extracted CMC and AgNPs@CMC materials (Figure 4) in the range of 2000–50 cm^−1^. There were two clear and intense peaks for the extracted CMC at ∼1395 cm^−1^ and 1710 cm^−1^, which could be due to the in-plane (CH, OH) vibrations and ν(CO) vibrations [50,51]. The shift of the CO band could be attributed to the interaction of the polymeric chains [52]. Obviously, the AgNPs’ incorporation into CMC changed the Raman spectroscopy characteristics, and more peaks became intense and clear, especially between 1000 and 1500 cm^−1^. This behavior could be explained by the following two reasons: First, the clear absorption peak around 970 nm was near the excitation laser wavelength (1064 nm), which could provide more intense Raman absorption [53]. Second, the morphology of the prepared Ag nanoparticles could facilitate the increase in the localized electric field. Both reasons could lead to an increase in the scattered Raman intensity. From the obtained Raman spectra, it could be concluded that AgNPs were successfully incorporated into and interacted with the extracted CMC.

#### 3.1.5. UV–VIS Spectroscopy

**Figure 5:**
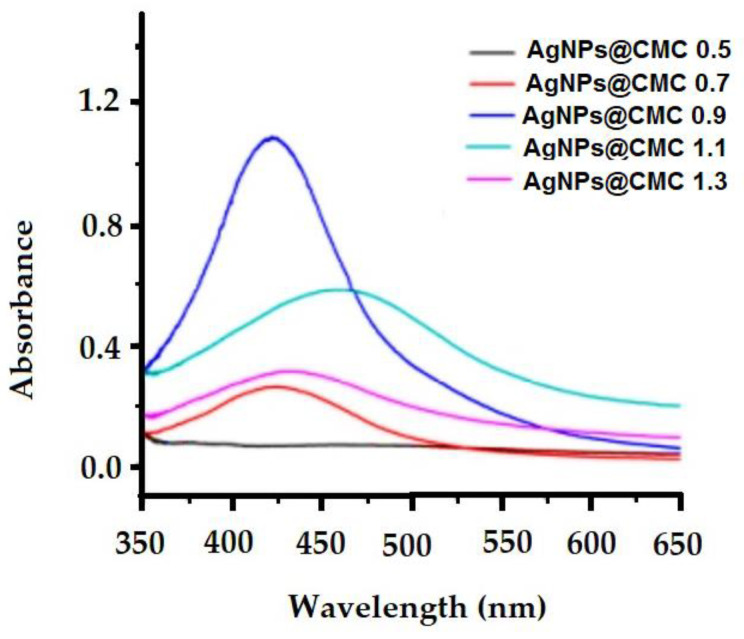
UV−VIS spectra of CMC extracts containing different AgNP contents.

The design of AgNPs at the CMC matrix was investigated by measuring UV–VIS spectroscopy (Figure 5). Figure 5 shows the effect of silver nanoparticle concentrations on the UV−VIS spectra of carboxymethyl cellulose extracts with different degrees of substitution (D.S.; 0.5–1.3) in the silver nanoparticle deposition process. In the measured UV–VIS spectroscopy, a broad peak around the wavelength of 420 nm with absorbance around 1.12 was seen, which could be assigned to the surface plasmon resonance (SPR) of the conducting electrons of the formed AgNPs. Additionally, the SPR band broadened with a clear shift from 420 to 450 nm, indicating an increase in particle size as the Ag content increased, which could be explained by the agglomeration of Ag particles. In short, UV–VIS spectroscopy indicates the existence of AgNPs via the peak of SPR at the surface of the extracted CMC.

#### 3.1.6. Thermal Analysis

**Figure 6:**
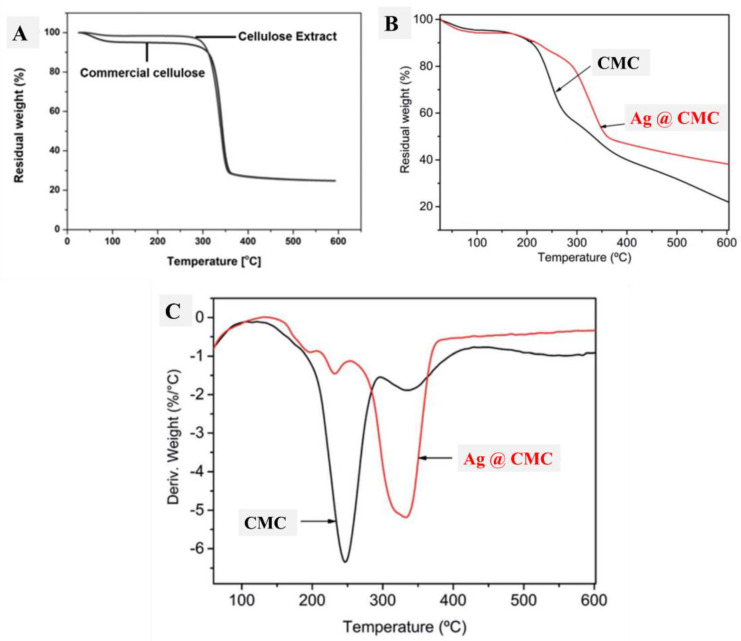
TGA of (**A**) commercial cellulose and cellulose extract, (**B**) TGA of CMC and AgNPs@CMC, and (**C**) DTG of CMC and AgNPs@CMC.

Thermal analysis (TGA) was performed to understand the thermal stability of the studied CMC materials. Figure 6A shows the TGA curves of extracted cellulose, which were compared with those of commercial cellulose. Interestingly, the thermal behavior of both extracted and commercial cellulose was the same after 300 °C, which indicates the successful extraction of cellulose. Below 300 °C, the extracted cellulose had nearly no loss, and commercial cellulose had a 6% loss because of the hydrated water of the commercial cellulose, which could be found in the case of extracted cellulose. Therefore, the extracted cellulose had better purity compared with commercial cellulose, which had more humidity, as seen from TGA (Figure 6A). After that, the formed CMC and its composite with AgNP materials were studied in terms of TGA and their differential curves (DTG), as displayed in Figure 6B and 6C, respectively. The first part of the TGA curve (less than 200 °C) was similar in both CMC and AgNPs@CMC, which indicates the similar moisture content in both CMC materials. Interestingly, decomposition of the CMC skeleton was seen at 260 °C and 340 °C, which confirms the interaction between the formed AgNPs and the CMC matrix, and this interaction provides more thermal stability to the formed material (AgNPs@CMC) [54]. Beyond 600 °C, the remaining contents were found at 22% and 38% for CMC and AgNPs@CMC, respectively. This behavior additionally indicates higher thermal stability in the case of AgNPs@CMC compared with the formed CMC.

### 3.2. Electrochemical Characteristics of AgNPs@CMC/GCE

**Figure 7:**
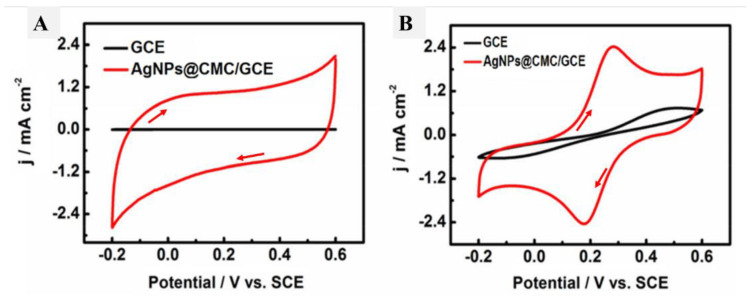
C–V curves of the GCE and AgNPs@CMC/GCE in (**A**) PBS (pH 9) and (**B**) 0.1 M KCl solution containing 5-mM [Fe(CN)_6_]^3−/4−^ at a scan rate of 50 mV s^−1^.

The electrochemical characteristics of the designed AgNPs@CMC were analyzed using a standard three-electrode cell system consisting of an AgNPs@CMC/GCE as the working electrode, a saturated calomel electrode (SCE) as the reference electrode, and a platinum wire as the counter-electrode. At the first electrochemical measurements, CV analysis was performed at a scan rate of 50 mV/s in the presence and absence of the designed AgNPs@CMC in PBS (pH 9) as a supporting electrolyte (Figure 7A). The measured C–V values of the investigated AgNPs@CMC in Figure 7A and the GCE did not have any observed redox peaks, which confirms the absence of any electrochemical activation, and this could be attributed to the chemistry of the electrode and electrolyte, which do not have any electroactive species to be easily oxidized or reduced. Interestingly, there was a sharp difference between the GCE and the electrode modified by AgNPs@CMC, because a higher current could be observed in the case of both anodic and cathodic directions. In traditional CV, the produced current has two parts: the faradaic one and the capacitive one [55]. The latter could be responsible for the higher current in the case of the electrode modified by AgNPs@CMC. In the absence of AgNPs@CMC, no current peaks could be observed; in addition, a negligible current was obtained compared with the AgNPs@CMC electrode. Additionally, the obtained current in the case of the AgNPs@CMC electrode was sharply higher than that of the GCE in the case of using a ferrocyanide/KCl electrolyte. This advancement could be a result of the enhanced electrical conductivity and active surface area of the modified electrode based on AgNPs@CMC compared with the pristine GCE. As the electroactive surface area is enhanced, the possible locations for accumulation of the negative or positive charged species go up and so increase the obtained capacitive current [56,57,58].

**Figure 8:**
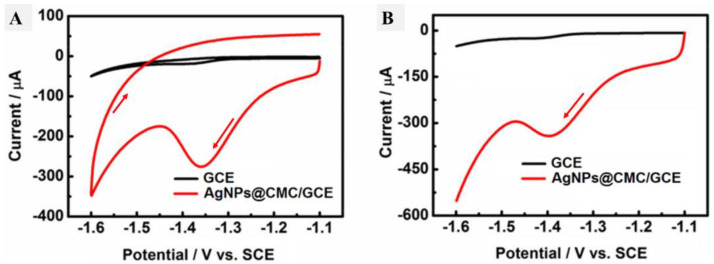
(**A**) C–V and (**B**) L–V curves of the GCE and AgNPs@CMC/GCE in PBS (pH 9) containing 30 mM HMF at a scan rate of 50 mV s^−1^.

**Figure 9:**
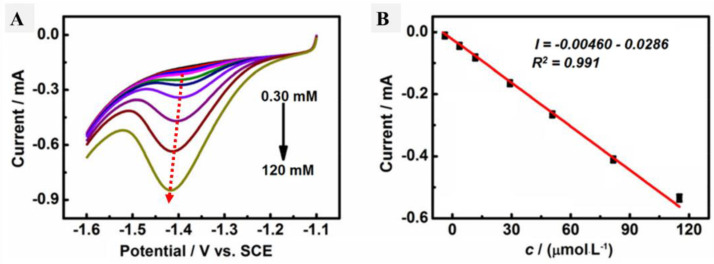
(**A**) LSV profiles at AgNPs@CMC/GCE in PBS (pH 9) containing different concentrations of HMF from 0.30 to 120 mM. (**B**) Relationship between the cathodic peak current and HMF concentration at a fixed scan rate of 100 mV s^−1^.

Cyclic voltammetry was performed in the presence of HMF in the electrolyte containing PBS (pH 9) for both the GCE and AgNPs@CMC/GCE, as shown in Figure 8A, and the linear scan voltammetry (LSV) results are shown in Figure 8B under the same conditions. In the case of the GCE, there were no reduction or oxidation peaks. In contrast, a cathodic peak was observed in the case of AgNPs@CMC/GCE at −1.35 V, and a corresponding cathodic current was seen around 300 µA. The existence of a clear cathodic peak could indicate the reduction of HMF molecules over the introduced AgNPs@CMC electrode. This behavior was confirmed by LSV, which had a clear cathodic peak around the same potential as CV. After that, LSV was repeated at AgNPs@CMC/GCE in PBS (pH 9) containing different concentrations of HMF from 0.30 mM to 120 mM at 100 mV/s, as shown in Figure 9A. In the sensing of HMF, high-purity nitrogen gas was used to purify the solution for 10 min before running voltammetry experiments and was kept flowing above the solution to maintain the nonoxygen environments. It could be observed that the cathodic peak was clear at all investigated concentrations of HMF and the measured cathodic current sharply improved with the increase in the molarity of HMF in the supporting electrolyte. AgNP-modified electrodes are known for their increased electroactive surface area and superior conductivity compared with unmodified electrodes [59]. They can detect various inorganic and organic analytes on the basis of the electrochemical responses of AgNPs [60,61]. The electrochemical mechanism is based on the interaction between HMF and AgNPs before the AgNPs lose these features during their conversion to Ag+. This conversion reduces the amount of AgNPs, which reflects the HMF concentration in the unknown solution. Therefore, there was a reduction peak of HMF molecules using AgNPs as an electrochemical working electrode. A pure polymer electrode has no electroactive peaks. Therefore, AgNPs were incorporated into the CMC polymer matrix. The relationship between the HMF content and the obtained cathodic current was studied, as displayed in Figure 9B. Linear behavior was characterized, with R2 equal to 0.991 and within the cathodic current range of 0.00460 mA–0.0286 mA. The linear behavior between the HMF concentration and the peak cathodic current could be used as a solid indicator to confirm the possibility of using the designed AgNPs@CMC electrode as a sensing electrode for HMF contents. HMF is reported to have a negative impact on human health, such as cytotoxicity toward mucous membranes, mutagenicity, or carcinogenicity toward humans or animals. HMF is limited to 40 mg/kg in general (except honey) and 80 mg/kg in honey declared to be from a tropical region. Fresh honey generally contains less than 15 mg/kg of HMF, but over 40 mg/kg is used to guarantee the honey has not undergone excessive heating during processing. Finally, the AgNPs@CMC-introduced electrode was applied to electrochemically determine the HMF content in real molasses Samples that were treated, as mentioned in the last paragraph of the experimental part, and the results are organized in Table 1. The content of HMF was found from 76 to 87 mg/g of the real sample, with a maximum SD of around 5.3%, which indicated the precision of the applied electrochemical method. In short, AgNPs@CMC could be used as a commercial electrode for HMF sensing.

## 4. Conclusions

A novel electrode for HMF sensing was introduced based on Ag nanoparticles in carboxymethyl cellulose (AgNPs@CMC). The prepared material was studied using different characterization tools to determine its morphology and structure. XRD data confirmed the successful design of cubic AgNPs (fm3m-225) in the CMC structure containing an amorphous part from CMC and a crystalline part from the AgNPs’ side. The morphology of the prepared Ag-CMC has a composite morphology between nanoparticles and a fibrous morphology. Therefore, the studied physicochemical techniques confirmed the incorporation of crystalline AgNPs into the CMC structure. HMF sensing was studied using voltammetric experiments at different HMF concentrations. The linear behavior between the HMF concentration and the peak cathodic current could be used as an indicator to confirm the possibility of using the designed AgNPs@CMC electrode as a sensing electrode for HMF contents.

## Figures and Tables

**Table 1:** Determination of HMF in real samples with AgNPs@CMC (n = 5).

Molasses Samples	AgNPs@CMC Electrode	
	HMF (mg/g)	SD (%)
1	87	5.3
2	81	3.01
3	76	4.0

## Data Availability

The raw/processed data generated in this work are available upon request from the corresponding author.
